# Accessory thymus in posterior mediastinum

**DOI:** 10.4103/0971-9261.44765

**Published:** 2008

**Authors:** Sushmita Bhatnagar, Rohit Pradhan, Pankaj Shastri, Pradeep Shenoy

**Affiliations:** Department of Pediatric Surgery, Bai Jerbai Wadia Hospital for Children, Parel, Mumbai - 400 012, India

**Keywords:** Accessory thymus, posterior mediastinum

## Abstract

Presence of thymus in the normal position as well as in the posterior mediastinum is an unusual phenomenon. We report here a case of posterior mediastinal mass in a 20-month old male child who presented with dysphagia and dry cough. Investigations revealed it to be a solid posterior mediastinal mass, suspected to be either lymphoma or a neuroblastoma. Excision of the mass followed by histopathologic examination revealed the mass to be a normal thymus. This case indicates that a benign mass, an accessory thymus, though rare, can be located in the posterior mediastinum and cause symptoms similar to solid mediastinal tumors.

## INTRODUCTION

Mediastinal masses are common in children. Approximately 30% occur in the anterior, 30% in the middle, and 40% in the posterior compartment of the mediastinum.[1] Accessory thymus body along the line of embryonic descent is common but not clinically significant (may be found in 25% of the population). Occurrence in the posterior mediastinum is rare, and clinical presentation is due to compression of the neighboring organs such as esophagus and trachea. Accessory thymus appears as a solid mass in the posterior mediastinum resembling neurogenic tumors, which comprise 90% of the posterior mediastinal masses in the pediatric age group. We describe here a case of posterior mediastinal mass that was suspected to be a neuroblastoma and proved to be a normal thymus on histopathology.

This case is unique as it presented with dysphagia, which is not a common mode of presentation described in the literature due to its predisposition to be on the right of the midline.[2] Tracheal compression is more commonly seen. Approximately 30 cases have been reported, some of which do not clearly differentiate accessory from ectopic thymus.

## CASE REPORT

**Figure 1:**
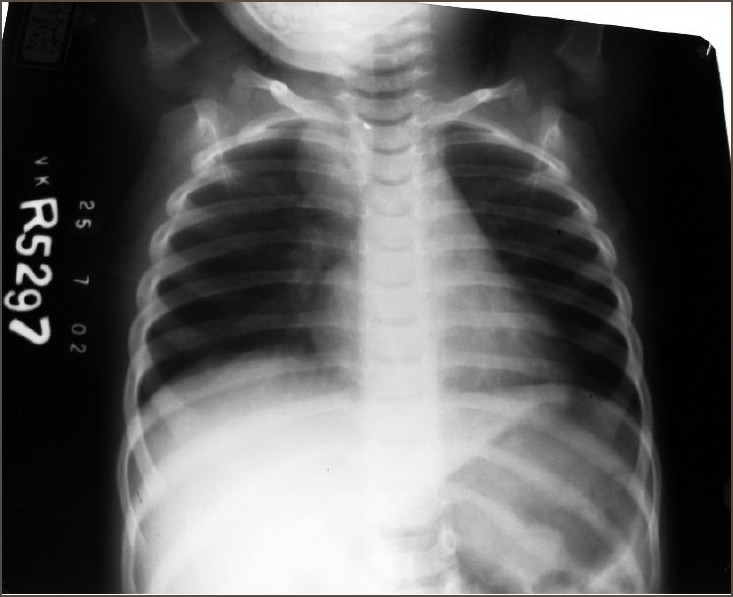
An X-ray chest showing a right paratracheal soft tissue density

A 20-month-old male child was admitted with complaints of dry cough, dysphagia, and low-grade fever of one-month duration. There was no abnormality detected on clinical examination. On investigating the child, his Hb was 10.8g%, WBC – 9,700, S. LDH – 356, LFT and RFT were normal. An x-ray chest revealed superior mediastinal widening [Figure 1]. HRCT chest showed a lesion in the posterior mediastinal region with mass effect on the esophagus and trachea and a normally placed thymus which was enlarged but normal for age. The most common differential diagnosis of this mass – neuroblastoma was suspected and child investigated for the same. All investigations including MIBG, Urinary VMA, and serum alpha-fetoproteins were normal. In view of the obstructive symptoms excision of the mass was planned. At right posterolateral thoracotomy through fourth intercostal space, a nodular mass was found behind the trachea extending up to the carina not communicating with any other structure. A normal thymus was found in the anterior mediastinum. The posterior mediastinal mass was completely excised after careful dissection of the trachea, esophagus and the nerves and sent for histopathology, which revealed normal thymic tissue.

Post-operatively, the child developed right phrenic nerve neuropraxia causing asymptomatic right eventration of diaphragm. The child was followed up for 4 years. An x-ray chest done 1 year later showed complete resolution of the eventration and the latest x-ray 6 months ago was also normal.

## DISCUSSION

Posterior mediastinal accessory thymus along with thymic tissue at the normal site cannot be satisfactorily explained by the normal embryologic development of the organ.[3] Posterior mediastinal accessory thymus is extremely rare.[4–9] However, details about the presence or absence of a normal thymus located in the normal position are not available.[9]

Pressure symptoms due to tracheal or esophageal compression due to thymic masses has been described by several authors as the most common mode of presentation.[10,11]

The accessory thymus tissue can appear in two forms.[8] It can be connected to the anterior normal thymus or could be completely free from it, as in our case. If it is communicating with the normal thymus anteriorly, the diagnosis can be established on table, but more often the diagnosis is established post-operatively on histopathology as in our case, with similar experiences from other authors who had excised the mass for suspected neoplasm.[4,9]

In scenarios where the child is asymptomatic, establishing a confirmatory diagnosis preoperatively is imperative as incidentally detected, asymptomatic accessory thymic masses need not be treated surgically. Several radiological and radionuclide modalities such as contrast-enhanced CT scan and CT-guided biopsy,[2] MRI,[8] and gallium scan[12] are available to arrive at a diagnosis. In children, normal thymic tissue is homogeneous and slightly more intense than muscle on T1-weighted MR images and slightly less intense or isointense relative to fat on T2-weighted images.[13] In spite of several characteristics on imaging, except for histopathology, none of these are confirmatory in the absence of continuation between the anterior and posterior thymus.[2] Several authors had to submit to histopathological diagnosis to rule out malignancy.[14,15] Recently, video-assisted thoracoscopy and biopsy has been reported as a tool to establish the diagnosis of a thymic mass.[16] Excision of the posterior mediastinal thymus is not mandatory unless it is causing obstructive symptoms,[8] especially in ectopic thymic tissue. In aberrant thymic masses, excision may be undertaken at any age without any risk to the patient.
